# Single‐centre experience of implementing physiotherapist‐led prehabilitation for chimeric antigen receptor T cell therapy

**DOI:** 10.1002/jha2.1006

**Published:** 2024-09-23

**Authors:** Orla McCourt, Paul Maciocia, Claire Roddie, Angela Hwang, Leigh Wood, Aikaterini Panopoulou, Deborah Ann Springell, Maise Al Bakir, Maeve O'Reilly

**Affiliations:** ^1^ Department of Haematology University College London Hospitals NHS Foundation Trust London UK; ^2^ Research Department of Haematology University College London Cancer Institute London UK

**Keywords:** chimeric antigen receptor T‐cell therapy, exercise, physiotherapy, rehabilitation

## Abstract

**Introduction:**

This report outlines the evaluation of physiotherapist‐led prehabilitation/rehabilitation for recipients of chimeric antigen receptor T (CAR‐T) cell therapy.

**Methods:**

A hybrid approach was used, incorporating in‐person assessment of quality of life and functional capacity (6‐min walk test and timed sit‐to‐stand test), and a personalised home exercise programme with remotely delivered physiotherapist support pre/post‐admission.

**Results:**

Functional deficits were prevalent at referral for CAR‐T. Prehabilitation and rehabilitation were highly acceptable to patients, and improvements in functional capacity were documented pre‐admission.

**Conclusion:**

This data highlights the importance of pre‐CAR‐T functional assessment and prehabilitation to optimise preparation and recovery.

## 1 INTRODUCTION

Autologous chimeric antigen receptor T‐cell therapies (CAR‐T) targeting CD19 have transformed treatment options for patients with relapsed/refractory B‐cell malignancies [1, 2]. Similarly to many cancer treatments, CAR‐T impacts quality of life (QOL), health status and functional capacity. During treatment, patients can experience physical, psychological and cognitive side effects which are compounded by the underlying disease and prior treatments [3]. Functional concerns in those undergoing CAR‐T are varied and deficits can be evident before, during and after treatment. Similar to patients undergoing stem cell transplantation (SCT), deconditioning and functional deficits are precipitated by lack of activity and consequences of prior treatment. Physical impairments and fatigue further result in reduced ability to undertake activities of daily living independently, and to engage socially and can have a negative effect on overall QOL during and beyond treatment.

Rehabilitation is considered a central element of cancer care and is becoming increasingly pertinent as for most cancers the incidence is increasing, but in parallel survival is improving and patients are surviving longer following their diagnoses [4]. Early rehabilitation or ‘*p*rehabilitation’ can 1) prepare people with cancer physically and mentally for their treatment, and 2) prepare them to commence restorative rehabilitation as soon as possible following treatment. Although there are no published trials of exercise or rehabilitation interventions in CAR‐T patients, positive effects on QOL and fatigue are evident among haematological cancer patients undergoing SCT and chemotherapy [5]. Limited literature exists exploring the rehabilitation needs of CAR‐T recipients but it has been suggested that exercise literature amongst allogeneic SCT recipients could be used to inform the implementation of prehabilitation and rehabilitation in CAR‐T pathways [6].

Generating specific data to define and better understand the rehabilitation needs of the expanding, diverse patient populations undergoing CAR‐T can inform multidisciplinary approaches to care. Integration of rehabilitation, including the promotion of supported self‐management, prepares patients better for treatment and facilitates recovery. As the application of CAR‐T is moving further up treatment lines and incorporating wider populations in clinical trials, greater emphasis on physically and psychologically optimising recipients in this ever‐changing care pathway is required to continue improving patient outcomes and resource utilisation.

This report describes the development, implementation and evaluation of a physiotherapy‐based prehabilitation and rehabilitation service embedded into the clinical pathway for CAR‐T at a large UK centre.

## 2 METHODS

A single centre service evaluation. Stakeholder activities with previous CAR‐T recipients and health care professionals involved in their care informed the design of the prehabilitation/rehabilitation service.

Patients attending outpatient clinics for consideration for CAR‐T underwent physiotherapy assessment including QOL questionnaires (EORTC QLQ‐C30 [7] and EQ5D‐5L [8]) and objective functional assessments (6‐min walk test [6MWT] and 1‐min sit‐to‐stand test [1 min‐STS]). The predicted 6MWT distance was calculated using a reference equation [9] and baseline scores were calculated as a percentage of the predicted score. Reference values for the 1 min‐STS were used for comparison to expected values for age and sex [10]. A baseline assessment was undertaken during the first CAR‐T clinical appointment and a follow‐up assessment was prior to hospital admission for CAR‐T. QOL was assessed again at three months post‐CAR‐T.

Prehabilitation and rehabilitation input involved personalised support to increase aerobic physical activity and provision of a tailored strengthening home exercise programme (incorporating upper and lower limb, multi‐joint exercises, using bodyweight, and/or resistance bands). Written support was provided with an intervention booklet including advice on managing fatigue, pain and behaviour change (e.g., identifying barriers/facilitators, goal setting, and log sheets). In addition, onward referral to community rehabilitation and other support services were made where required, particularly post‐CAR‐T.

Patients were supported with regular telephone input from the physiotherapist in the prehabilitation phase to guide the progression of home exercise. During hospital admission, patients received regular input from ward‐based therapists. Rehabilitation input started on discharge from CAR‐T admission, with patients offered 1–2 weekly telephone input to guide recovery until 3 months post‐CAR‐T infusion.

Participation in physiotherapy assessments and telephone calls was optional. All data was anonymised for analysis. On completion of physiotherapy input, patients were asked to complete an anonymous online patient experience survey. Data was summarised descriptively (median, range, frequencies, and proportions). Paired t‐tests were used to assess pre‐post outcomes. A *p*‐value of < 0.05 was used to indicate statistical significance.

## 3 RESULTS

Over four consecutive months (June–September 2023), 22 patients were assessed at baseline in the CAR‐T clinic at the point of referral for CAR‐T. Of these, 19 (86%) patients were admitted and treated, two (9%) had progressive disease and did not receive CAR‐T and one (5%) patient did not receive CAR‐T within the timeframe of this project.

Eighteen patients underwent both a baseline and pre‐admission assessment. The median age was 61 years and the majority received axicabtagene ciloleucel for relapsed diffuse large B‐cell lymphoma (DLBCL). Demographics and clinical features are summarised in Table 1.

**TABLE 1:** Demographics and clinical features.

	(*n* = 18)
Age median [IQR]	61 [48, 62.8]
range	37–74
Sex male *n* (%)	10 (56%)
Diagnosis *n* (%)	
DLBCL	14 (78%)
DLBCL (transformed follicular)	2 (11%)
DLBCL (transformed WM)	1 (6%)
Mantle Cell Lymphoma	1 (6%)
Ethnicity n(%)	
White or White British	14 (78%)
Other White background	1 (6%)
Other Black background	1 (6%)
Other ethnic group	1 (6%)
Unknown	1 (6%)
Employment Status *n* (%)	
Retired	10 (56%)
Full‐time employment	5 (28%)
Unemployed	1 (6%)
Self‐employed	1 (6%)
Volunteer work	1 (5%)
Bridging Therapy *n* (%)	
RBP/R‐Pola	10 (56%)
RT	2 (11%)
RBP/R‐Pola + RT	2 (11%)
RGDP	1 (6%)
RGDP + RT + R‐Pola	1 (6%)
R‐CHOP	1 (6%)
None	1 (6%)
CAR‐T Product Received *n* (%)	
Axicabtagene ciloleucel	16 (89%)
Tisagenlecleucel	1 (6%)
Academic product on clinical trial	1 (6%)
Admission Total LOS median [IQR], days	34 [33, 35]
LOS D0 to discharge median [IQR], days	29 [28, 31]
Prehabilitation Input median [IQR]	
Number of contacts/patient	2.5 [1.3, 3]
Duration of contacts, minutes	30 [20, 30]
Rehabilitation Input median [IQR]	
Number of contacts/patient	3 [2, 3]
Duration of contacts, minutes	25 [20. 30]

Abbreviations: DLBCL, diffuse large B‐cell lymphoma; IQR, inter‐quartile range; LOS, length of stay; RBP, rituxamab‐bendamustine‐polatuzumab; R‐CHOP, rituximab‐cyclophosphamide‐doxorubicin‐vincristine; RGDP, rituximab‐gemcitabine‐dexamethasone‐cisplatin; R‐Pola, rituximab‐polatuzumab; RT radiotherapy; WM, Waldenstrom's macroglobulinemia.

### 3.1 Functional outcomes

The median baseline 6MWT distance was 450 m (*n* = 17, inter‐quartile range [IQR] 374.6, 505.4). 94% of participants walked < 80% of their predicted 6MWT distance with a group median predicted distance of 67.1%. The baseline median 1 min‐STS was 19.5 repetitions (*n* = 16, IQR 16.8, 21.5). All patients scored below average (< 25th centile) 1 min‐STS and 69% performed < 2.5th centile expected score for their age and sex [10].

**FIGURE 1:**
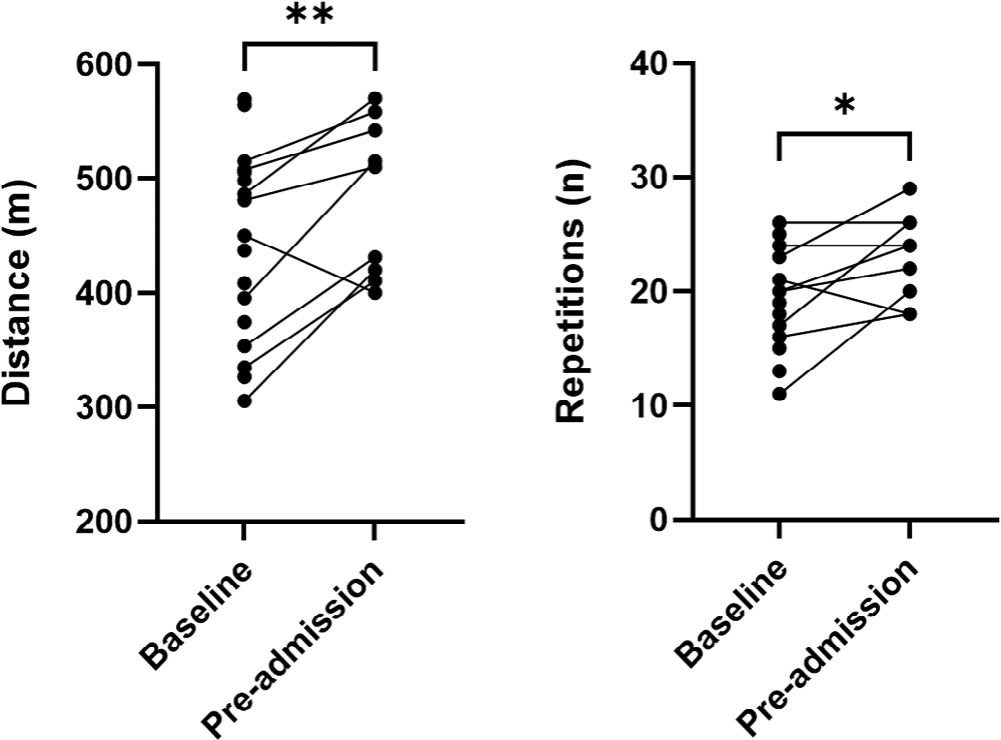
Changes in functional tests: 6‐min walk test distance; 1‐min timed sit‐to‐stand test repetitions (*p < 0.05 and **p < 0.01).

Data for pre‐ and post‐prehabilitation functional test outcomes were available for nine of 18 (50%) patients (Figure 1). Reasons for no post‐prehabilitation functional outcomes included four (22%) were missing due to lack of physiotherapist cover, three (17%) lost to follow‐up, one (6%) patient had disease symptoms that contraindicated testing, and one (6%) declined to undergo testing. There were no significant differences in patient characteristics or baseline functional outcomes between those included in the functional outcomes analysis and those who were not. The median time between baseline assessment and pre‐TCI follow‐up was 5 weeks (IQR 5, 6.1). The median time between baseline assessment and admission date was 6.5 weeks (IQR 5.4, 9.4).

There was a significant improvement in 6MWT distance (+58.6 m, 95% confidence interval [CI] 18.7, 98.6; *p* = 0.01) following prehabilitation.Overall, 78% of patients had a clinically meaningful change in 6MWT. 1‐min‐STS score improved +3.2 repetitions (95% CI 0.03, 6.4, *p* = 0.05) following prehabilitation, with44% of patients demonstrating a clinically meaningful increase in 1 min‐STS.

### 3.2 Quality of life

There were no statistical or clinically important differences in median scores of the EORTC C30. There was no difference in the proportions of participants reporting moderate or severe problems with the EQ5D between baseline and admission (Supporting Information).

Of the 18 patients who underwent CAR‐T, electronic QOL questionnaires were requested for remote completion in those who were in remission at 3 months post‐CAR‐T. Of the 72% of patients in remission, only 54% (*n* = 7) completed the measures which limited analysis of change post‐CAR‐T.

### 3.3 Patient experience

An anonymous online survey was sent via email to 15 patients after their final contact with the physiotherapist, approximately 3 months post‐CAR‐T. Nine (60%) responses were received. 89% reported that receiving prehabilitation support before CAR‐T was of great importance in preparing them for CAR‐T/hospital admission. 89% reported rehabilitation support after CAR‐T was important in assisting their recovery.

78% agreed or strongly agreed that the intensity of their exercise programme was appropriate; that the physiotherapist worked with them to tailor their programme to their fitness level; and that the exercise programme was beneficial to them. 89% of respondents strongly agreed that additional input from the physiotherapy service supported their self‐management of their overall health and well‐being whilst undergoing CAR‐T.

## 4 DISCUSSION

This brief report outlines an innovative approach to integrating prehabilitation into the CAR‐T pathway at a large UK centre. Notably, the incorporation of functional assessment at referral for CAR‐T indicates a high prevalence of impaired functional capacity in most patients. Nearly all patients walked < 80% of their predicted 6MWT distance and more than two‐thirds performed < 2.5th centile expected 1 min‐STS, both indicating significantly impaired fitness and leg strength [10].

Hybrid prehabilitation input, including objective face‐to‐face assessment with regular remote physiotherapy input, was well received by patients and improvements in functional measures indicate promise. The significant improvement in 6MWT was greater than its minimally important difference of 30.5 m [11]. Improvement in 1 min‐STS is considered in line with a minimally important difference for this outcome [12, 13]. These clinically important improvements in functional outcomes suggest prehabilitation can have a significant role in optimising functional capacity prior to admission for CAR‐T. In contrast to changes reported in previous clinical trials [14], we did not see any change in QOL with prehabilitation input within the timepoints assessed.

The findings from this single‐centre service evaluation, although insightful, have several limitations. The small sample size, due to time‐scale limitations of project funding, limits generalisability to the wider population of CAR‐T recipients. The geographical spread of patients from the treatment centre limited the ability to capture objective follow‐up data post‐CAR‐T but emphasised the important need for, and acceptability of, remotely capturing electronic PROM data to assess QOL following treatment.

Exercise interventions are known to improve functional capacity and QOL in patients undergoing allogeneic SCT [5] and this real‐world evaluation indicates an extension of benefit to those preparing for CAR‐T. Despite growing evidence for rehabilitation and exercise interventions in different haematological populations and the recognised requirement to have access to rehabilitation services in haematological cancer services in the UK [15], the incorporation of specialist allied health professionals, such as physiotherapists, embedded within multidisciplinary teams to deliver rehabilitation remains rare, even in specialist cancer centres [16]. As the number of indications for cellular therapies such as CAR‐T continues to expand, approaches to optimise and streamline the model of care in the real world will become increasingly important and the provision of rehabilitation must be prioritised to meet the needs of patients.

## AUTHOR CONTRIBUTIONS

Orla McCourt and Maeve O'Reilly designed the project. Orla McCourt collected and analysed the data. Orla McCourt and Maeve O'Reilly wrote the manuscript. All authors reviewed and approved the final manuscript.

## CONFLICT OF INTEREST STATEMENT

Claire Roddie: Advisory boards/honoraria Autolus, BMS, Janssen, Kite, Novartis. Angela Hwang: Honoraria Kite/Gilead. Aikaterini Panopoulou: Travel support from Kite. Maeve O'Reilly: Honoraria Janssen, Kite, Novartis. Advisory board Autolus, Kite. Travel grant Kite. Orla McCourt and Maeve O'Reilly jointly obtained an educational grant via the Gilead UK and Ireland Fellowship Programme (grant ID 17617) to undertake this work.

## ETHICS STATEMENT

In line with Health Research Authority (HRA) guidance, ethical approval was not required based on the HRA decision tool. Local governance procedures for quality improvement work were followed. Informed consent was gained in line with clinical practice guidance, and implied consent was obtained when patients returned the completed outcome measures.

## PATIENT CONSENT STATEMENT

The authors have confirmed patient consent statement is not needed for this submission.

## CLINICAL TRIAL REGISTRATION

The authors have confirmed clinical trial registration is not needed for this submission.

## Supporting information



Supporting Information

## Data Availability

The datasets used and/or analysed during the current study are available from the corresponding author upon reasonable request.

## References

[1] Schuster SJ , Bishop MR , Tam CS , Waller EK , Borchmann P , McGuirk JP , et al. Tisagenlecleucel in adult relapsed or refractory diffuse large B‐cell lymphoma. N Engl J Med. 2019;380(1):45–56. 30501490 10.1056/NEJMoa1804980

[2] Neelapu SS , Locke FL , Bartlett NL , Lekakis LJ , Miklos DB , Jacobson CA , et al. Axicabtagene Ciloleucel CAR T‐cell therapy in refractory large B‐Cell lymphoma. N Engl J Med. 2017;377(26):2531–2544. 29226797 10.1056/NEJMoa1707447 PMC5882485

[3] Whisenant MS , Srour SA , Williams LA , Subbiah I , Griffin D , Ponce D , et al. The unique symptom burden of patients receiving CAR T‐Cell therapy. Semin Oncol Nurs. 2021;37(6):151216. 34629213 10.1016/j.soncn.2021.151216

[4] Robb KA , Davis J . Examining progress in cancer rehabilitation: are we closer to parity of esteem? Eur J Cancer Care (Engl). 2015;24(5):601–604. 26303585 10.1111/ecc.12369

[5] Abo S , Denehy L , Ritchie D , Lin KY , Edbrooke L , McDonald C , et al. People with hematological malignancies treated with bone marrow transplantation have improved function, quality of life, and fatigue following exercise intervention: a systematic review and meta‐analysis. Phys Ther. 2021;101(8). 10.1093/ptj/pzab130 33989413

[6] Obaisi O , Fontillas RC , Patel K , Ngo‐Huang A . Rehabilitation needs for patients undergoing CAR T‐Cell therapy. Curr Oncol Rep. 2022;24(6):741–749. 35267151 10.1007/s11912-022-01240-0 PMC8907385

[7] Aaronson NK , Ahmedzai S , Bergman B , Bullinger M , Cull A , Duez NJ , et al. The European Organization for Research and Treatment of Cancer QLQ‐C30: a quality‐of‐life instrument for use in international clinical trials in oncology. J Natl Cancer Inst. 1993;85(5):365–376. 8433390 10.1093/jnci/85.5.365

[8] Pickard AS , De Leon MC , Kohlmann T , Cella D , Rosenbloom S . Psychometric comparison of the standard EQ‐5D to a 5 level version in cancer patients. Med Care. 2007;45(3):259–263. 17304084 10.1097/01.mlr.0000254515.63841.81

[9] Gibbons WJ , Fruchter N , Sloan S , Levy RD . Reference values for a multiple repetition 6‐minute walk test in healthy adults older than 20 years. J Cardiopulm Rehabil. 2001;21(2):87–93. 11314289 10.1097/00008483-200103000-00005

[10] Strassmann A , Steurer‐Stey C , Lana KD , Zoller M , Turk AJ , Suter P , et al. Population‐based reference values for the 1‐min sit‐to‐stand test. Int J Public Health. 2013;58(6):949–953. 23974352 10.1007/s00038-013-0504-z

[11] Bohannon RW , Crouch R . Minimal clinically important difference for change in 6‐minute walk test distance of adults with pathology: a systematic review. J Eval Clin Pract. 2017;23(2):377–381. 27592691 10.1111/jep.12629

[12] Vaidya T , de Bisschop C , Beaumont M , Ouksel H , Jean V , Dessables F , et al. Is the 1‐minute sit‐to‐stand test a good tool for the evaluation of the impact of pulmonary rehabilitation? Determination of the minimal important difference in COPD. Int J Chron Obstruct Pulmon Dis. 2016;11:2609–2616. 27799759 10.2147/COPD.S115439 PMC5079690

[13] Crook S , Busching G , Schultz K , Lehbert N , Jelusic D , Keusch S , et al. A multicentre validation of the 1‐min sit‐to‐stand test in patients with COPD. Eur Respir J. 2017;49(3). 10.1183/13993003.01871-2016 28254766

[14] Patrick DL , Powers A , Jun MP , Kim Y , Garcia J , Dehner C , et al. Effect of lisocabtagene maraleucel on HRQoL and symptom severity in relapsed/refractory large B‐cell lymphoma. Blood Adv. 2021;5(8):2245–55. 33904895 10.1182/bloodadvances.2020003503 PMC8095132

[15] National Institute for Health and Clinical Excellence . Haematological cancers: improving outcomes. NICE guideline [NG47]. London: National Institute for Health and Clinical Excellence; 2020. https://www.nice.org.uk/guidance/ng47/resources/haematological‐cancers‐improving‐outcomes‐pdf‐1837457868229 27280275

[16] Miller J , Barrett R , Canning P , Taggart S , Young V . Multi‐professional management within haemato‐oncology. In: Rankin J , Roob K , Murtagh N , Cooper J , Lewis S , eds. Rehabilitation in cancer care. Oxford: Wiley‐Blackwell; 2008. pp. 113–130.

