# Supplementary material for: Single‐centre experience of implementing physiotherapist‐led prehabilitation for chimeric antigen receptor T cell therapy
Source: EJHaem. 2024 Sep 23;5(5):1033–7. doi: 10.1002/jha2.1006 (PMC11481005; doi:10.1002/jha2.1006)
Supplement: Supplementary file 1 — Supporting Information [file JHA2-5-1033-s001.docx]

**Table and Figure for Supplementary material**

Table S1 Quality of Life scores across timepoints

| Median [IQR] | Baseline  (n=18) | Preadmission  (n=18) | 3 Months post CAR-T  (n=7) |
| --- | --- | --- | --- |
| EORTC QLQ C30 Summary Score | 76.2 [67.0,82.3] | 80.0 [62.0,89.2] | 82.8 [69.4,89.6] |
| EORTC QLQ C30 domains |  |  |  |
| Global QOL | 62.5 [43.6,79.2] | 58.3 [50.0,75.0] | 66.7 [33.3,83.3] |
| Physical Functioning | 73.3 [68.3,86.7] | 80.0 [68.3,91.7] | 73.3 [60.0,83.3] |
| Role Functioning | 66.7 [50.0,83.3] | 66.7 [33.3,83.3] | 66.7 [50.0,91.7] |
| Emotional Functioning | 83.3 [60.4,91.7] | 79.2 [60.4,91.7] | 83.3 [70.8,87.5]] |
| Social Functioning | 66.7 [37.5,79.2] | 66.7 [33.3,79.2] | 66.7 [33.3,83.3] |
| Fatigue | 34.5 [25.0,44.0] | 35.0 [20.0,44.8] | 22.2 [22.2,50.0] |
| Pain | 16.7 [0.0,58.3] | 16.7 [0.0,45.8] | 33.3 [8.3,33.3] |


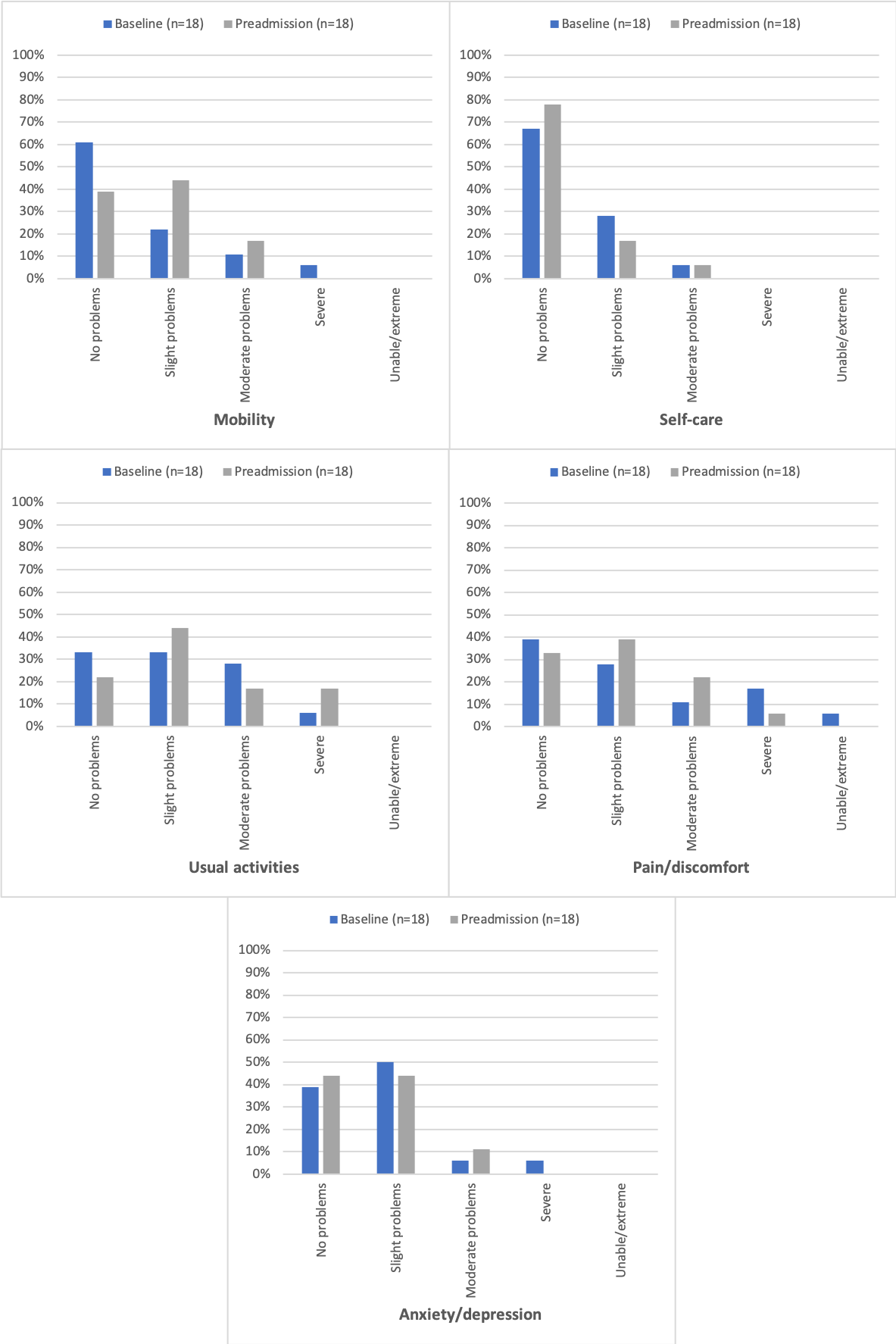


Figure S1 Proportion of responses by level of severity for EQ-5D-5L dimensions at baseline, pre-admission for CAR-T.
